# Bioinformatics-driven discovery of novel EGFR kinase inhibitors as anti-cancer therapeutics: *In silico* screening and *in vitro* evaluation

**DOI:** 10.1371/journal.pone.0298326

**Published:** 2024-04-16

**Authors:** Awwad A. Radwan, Fars Alanazi, Abdullah Al-Dhfyan

**Affiliations:** 1 Department of Pharmaceutics, Kayyli Chair for Pharmaceutical Industries, College of Pharmacy, King Saud University, Riyadh, Saudi Arabia; 2 Department King Faisal Specialized Hospital and Research Center, Cell Therapy & Immunobiology, Riyadh, Saudi Arabia; Kafrelsheikh University Faculty of Pharmacy, EGYPT

## Abstract

Epidermal growth factor receptor EGFR inhibitors are widely used as first line therapy for the treatment of non-small-cell lung cancer (NSCLC) in patients harboring EGFR mutation. However, the acquisition of a second-site mutation (T790 M) limited the efficacy and developed resistance. Therefore, discovery and development of specific drug target for this mutation is of urgent needs. In our study we used the ChemDiv diversity database for receptor-based virtual screening to secure EGFR-TK inhibitors chemotherapeutics. We identified four compounds that bind to the ATP-binding region of the EGFR-TK using AutoDock 4.0 and AutoDock Vina1.1.2 and post-docking investigations. The ligand showed hydrophobic interactions to the hydrophobic region of the binding site and engaged in hydrogen bonding with Met793. The ligands also explored π–cation interactions between the π-system of the ligand–phenyl ring and the positive amino group of Lys745. Molecular mechanics Poisson–Boltzmann surface area MM/PBSA per-residue energy decomposition analyses revealed that Val726, Leu792, Met793, Gly796, Cys797, Leu798, and Thr844 contributed the most to the binding energy. Biological evaluation of the retrieved hit compounds showed suppressing activity against EGFR auto phosphorylation and selective apoptosis-induced effects toward lung cancer cells harboring the EGFR L858R/T790M double mutation. Our work anticipated into novel and specific EGFR-TKIs and identified new compounds with therapeutic potential against lung cancer.

## Introduction

**Fig 1:**
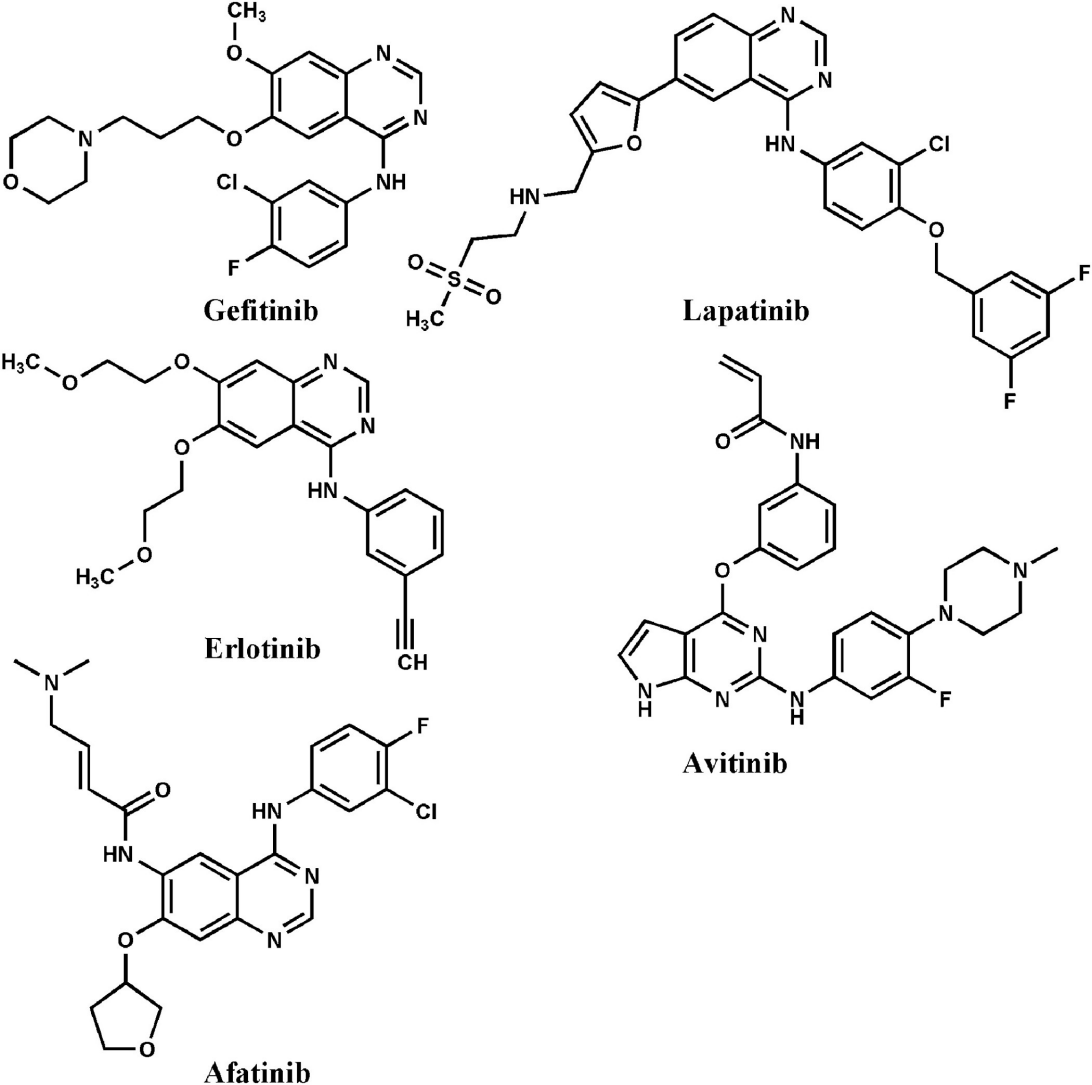
Representative chemical structure of epidermal growth factor receptor (EGFR) kinase inhibitor drugs.

The family of epidermal growth factor receptor tyrosine kinase (ErbBs) is an essential component of the cellular signaling pathways that control vital processes such as cell survival, differentiation, proliferation, and apoptosis [1]. The erythroblastic leukaemia viral oncogene, for which the receptors are identical, is the source of the ErbB family’s name. The four structurally conserved members of this family are epidermal growth factor receptors EGFR/ErbB1, ErbB2, ErbB3, and ErbB4. Their common domain structure includes an intracellular area with a juxtamembrane domain (53 aa), tyrosine kinase (TK) domain (260 aa), C-terminal tyrosine-rich region (232 aa). The extracellular domain that binds ligands includes a hydrophobic transmembrane segment and an extracellular segment [2, 3]. The growth factors (transforming growth factor-α (TGF-α) and epidermal growth factor (EGF)) bind to the extracellular portion of the receptor. This results in activation and phosphorylation of the TK domain at its C-terminal residues, causing the receptor to homo- and/or heterodimerize and initiate downstream signaling cascades [4]. EGFR is overexpressed in approximately 60% of individuals with non-small cell lung cancer (NSCLC), the most prevalent type of lung cancer and the primary cause of cancer-related deaths globally [5]. Clinical studies had highlighted EGFR dysregulation as a therapeutic target in NSCLC [6]. Four different types of anti-EGFR chemotherapies are currently available: monoclonal antibodies (mAbs), which target the extracellular domain of EGFR [7]; antisense oligonucleotides, which stop the synthesis of EGFR; antibody-based immunoconjugates [8, 9]; and small molecular-weight compounds that block tyrosine kinase activity. First-generation EGFR-TK inhibitors (EGFR-TKIs) includes gefitinib (Iressa^TM^, AstraZeneca), erlotinib (Tarceva^TM^, OSI-Pharma/Genentech/Roche), and lapatinib (Tykerb^TM^, GlaxoSmithKline) are small molecules that compete with ATP in the TK binding domain [10] (Fig 1). Second- and third-generation EGFR-TKIs include afatinib (Gilotrif^TM^; Boehringer Ingelheim) and avitinib (clinical trials), respectively. Although only a few NSCLC cases are gefitinib-susceptible, responses to treatment with gefitinib may be linked to various mutations in the EGFR-TK domain, including the L834R mutation, which increases kinase activity. However, there is evidence that patients with NSCLC with the L834R mutation as the primary cause of the disease can develop acquired resistance to gefitinib and erlotinib, resulting in the T766M mutation.

To date, 26 crystal structures of EGFR-TK, including its wild-type and mutant forms, have been described. These structures show that EGFR-TK exists in active and inactive conformers, which differ in terms of activation loop (A-loop) organization, Asp-Phe-Gly (DFG) motif, L834 and L837, and α-helix-C orientation [11]. With the aid of the X-ray structures of EGFR-TK bound to erlotinib or gefitinib, important binding interactions of quinazoline moiety to the kinase hinge region have been investigated [12, 13]. These investigations aid in understanding the relationship between EGFR-TK and its inhibitors that is principle in developing novel target-specific kinase suppressors. Target-based virtual screening (VS) is a high-throughput *in silico* drug-discovery approach [14]. Macromolecule-based VS utilizes a molecular docking technique that aids understanding of the three-dimensional (3D) structure of the target protein binding site. In numerous instances including those involving break-point cluster region-abelson murine leukemia (BCR-ABL) TK [15], checkpoint kinase 1(Chk1) [16], FK506 binding protein (FKBP) [17], and protein tyrosine phosphatases [18], structure-based VS methods have been helpful in identifying new inhibitors. A few EGFR-TK receptor-based VS studies against industrial and commercial chemical compounds in the anilinoquinazoline, pyridopyrimidine, and pyrrolopyrimidine families have been described [19].

In our study, we applied the VS study using EGFR-TK receptor-gefitinib complex structure against a diversity set of 350,000 small-molecule compounds (ChemDiv Library DC0, accessed Sep 08, 2022) in order to identify new EGFR inhibitors as potential anti-cancer agents. The process includes ligand- and structure-based pharmacophore mapping, molecular docking approaches in conjunction with post-docking analysis, molecular dynamics (MD) simulations, and molecular mechanics/generalized born surface area calculations (MM/GBSA). The study shed light on the mechanism of EGFR-TK enzyme suppression in four compounds. The EGFR-TK conformational changes observed in the MD simulation and the binding free energies of the compounds, as calculated using the MM/GBSA analysis explored the importance of the hydrophobic and electrostatic characteristics of the ligands for ligand-protein bindings.

## Methods and procedures

### Preparation of EGFR protein structure and compound dataset

The Protein Database (http://www.pdb.org (accessed 23-Dec-2021) was used to obtain the crystal structure of the EGFR-TK–gefitinib complex (PDB:4wkq) [20]. Missing residues were fixed and the entire protein sequence structure was numbered 694–1020, and the protein sequence structure was modified using Modeler10.4 software by ignoring the three terminal amino acids 1, 1021, and 1022. The structures of the screening library of 350,000 compounds were obtained from the ChemDiv (https://www.chemdiv.com/) (accessed on 1-Jan-2022) online collection using the publicly available discovery chemistry (DC01).

### Ligand-based VS

For drug-like chemical libraries, Ghose et al. recommended qualifying range with the following restrictions: molecular weight between 160 and 480; estimated logP between 0.4 and 5.6; molar refractivity between 40 and 130; and overall atom count between 20 and 70 [21]. A customized Ghose rule was used in screening the dataset using Mwt range of 350–480 and logP 2.5–4.8. LigandScout4.1 [22] was used for structure- and ligand-based database screening on the reduced library subset of 157,850 chemicals. LigandScout extracted gefitinib and its macromolecular environment from 4wkq PDB files and automatically created a 3D pharmacophore model. In addition, LigandScout was used to generate pharmacophore models based on a small-molecule database injected into the active site and produce docking pose results for VS at the binding site.

### Structure-based VS

Removing the heteroatoms allowed for the preparation of the receptor with the 3D crystallographic structure (PDB: 4wkq). Using AutoDock Vina1.1.2 [23], Autodock 4 [24] and LigandScout program-output library was scored and ranked. Docking process was validated using co-crystallized ligands (xray ligand). The root-mean-square deviation (RMSD) of the xray ligand-structure from its docked conformation was used as a measure in validating the docking protocols. The atuotodock4 program grid box dimensions were x = 11.138, y = 10.5, and z = 10.5 Å. The atuotodock-vina program grid box dimensions were x = 15.529, y = 14.712, and z = 17.414 Å and exhaustiveness value of 8. The atomic affinity potentials generated on a grid were used to measure the binding energy at each stage of the docking simulation. Using PyMOL software, the compounds’ binding conformations were visualized [26].

### MD simulations

Using AmberTools22 package [27] on ubuntu20.04, EGFR-TK-docked ligand complex of the hit compounds ZINC21802765, ZINC21802749, ZINC21802742 or ZINC21802768 was used as initial structures to the MD simulations process. A water box, 10 Å of TIP3P water model, was applied. The particle mesh Ewald was used to calculate long-range electrostatic interactions with the periodic boundary condition imposed. The ff14SB force field, and non-bonding interactions cut-off value 8 Å were assigned [28]. Counter ions and water molecules underwent 1000-cycle minimizations followed with 1000-cycle minimizations of the whole system. At first, the system equilibrated within a simulation time-period of 170 ps. During the first 20 ps, counter ions and water molecules equilibrated while the solutes kept restrained. During the next 50 ps, the amino acid side chains were relaxed and during the last 100 ps the whole system constraints were released. The MD simulations run for 10 ns at 298.15 K and 1 atm pressure using time step of two femtoseconds (fs). Throughout the simulations, 1 ps time-intervals were used to save the atom coordinates in the complex system. The starting structures from the MD simulation were utilized as the reference structures to calculate RMSDs using the CPPTRAJ module of AmberTools18 package to confirm the convergence of the MD simulation procedures. The local flexibility at each amino acid residue could be calculated using root-mean-square fluctuations (RMSFs) and reference structures that represented as average structures over the course of the last four ns trajectories.

### Binding free energy estimation

The EGFR-TK-ligand binding free energy (ΔG_binding_) was calculated by the MM/GBSA module [34] using 100 snapshots of the trajectories obtained during the last 4 ns simulation time. The input variables were set for the generalized born method igb = 5, salt concentration saltcon = 0.1 M and the level of output variable verbose = 1.

### Per-residue free energy decomposition

Molecular mechanics Poisson–Boltzmann surface area MM/PBSA.py was utilized to realize free binding energy calculations and to execute energy decomposition analysis, using the Poison-Boltzmann (PB) model from 100 snapshots during the last 4 ns simulation time [23]. The input variables for the Poisson Boltzmann variables, ionic strength istrng = 0.1 M, internal dielectric constant indi = 1.0, nonpolar optimization method inp = 1. The variables for the energy decomposition idecomp = 1 and the level of decomp_output dec_verbose = 2.

### Biological evaluations

#### Materials and cell preparations

The American Type Culture Collection (Rockville, MD, USA) provided EGFR wild-type lung adenocarcinoma A549 cells and EGFR L858R/T790M double mutant NSCLC H1975 cells. The cells were cultured in 75 or 150 mL tissue culture flasks at 37°C in a 5% CO_2_ humidified atmosphere with Dulbecco’s modified Eagle’s medium (DMEM), phenol red, 10% fetal bovine serum, 200 μM L-glutamine, and 1X antibiotic–antimycotic. For immunofluorescence and flow cytometry, the cells were plated in cell culture plates using DMEM. The stock solutions of all compounds ZINC21802765, ZINC21802749, ZINC21802742 and ZINC21802768 were prepared in DMSO, with the DMSO concentration in each treatment not exceeding 0.1% (v/v).

#### Immunofluorescence assay

H1975 cells were grown for 7 d on glass slides at a density of 20,000 cells/mL before being preserved in 4% formaldehyde. Compound ZINC21802765, ZINC21802749, ZINC21802742 or ZINC21802768 was added to H1975 cells. Following primary antibody staining for the cell signaling molecules EGFR and p-EGFR, fixed cells were next labeled with FITC-conjugated secondary antibodies and 1 μg/mL 4′-6-diamidino-2-phenylindole (DAPI), a fluorescent stain for nuclear DNA. Each antibody was added to each sample in triplicate. A BD Pathway 855 Bioimager (BD, Franklin Lakes, NJ, USA) was used to analyze the fluorescence staining intensity and intracellular localization (v/v).

#### Apoptosis assay

By employing a flow cytometer and the Vybrant apoptosis test kit (Annexin V, APC conjugate; Molecular Probes, Thermo Fisher Scientific, Waltham, MA, USA), the percentage of cells experiencing apoptosis in response to the tested compounds ZINC21802765, ZINC21802749, ZINC21802742 or ZINC21802768 and gefitinib, a reference medication, was calculated. A549 and H1975 cells that had been exposed to the test substances for 48 h were collected, pelleted, and resuspended in DMEM. A total of 104 cells were collected, stained with annexin V and DAPI to detect viability, and immediately examined on a BD LSRII Flow Cytometer (BD). If the cells tested negative for both Annexin V and DAPI, they were considered viable.

#### Statistical analysis

All results were presented as mean ± SEM. Student’s t-test using Excel^®^ was carried out to assess which treatment groups show significant differences from the control ones. The differences were considered significant when p < 0.05.

## Results and discussion

### Library establishment

**Fig 2:**
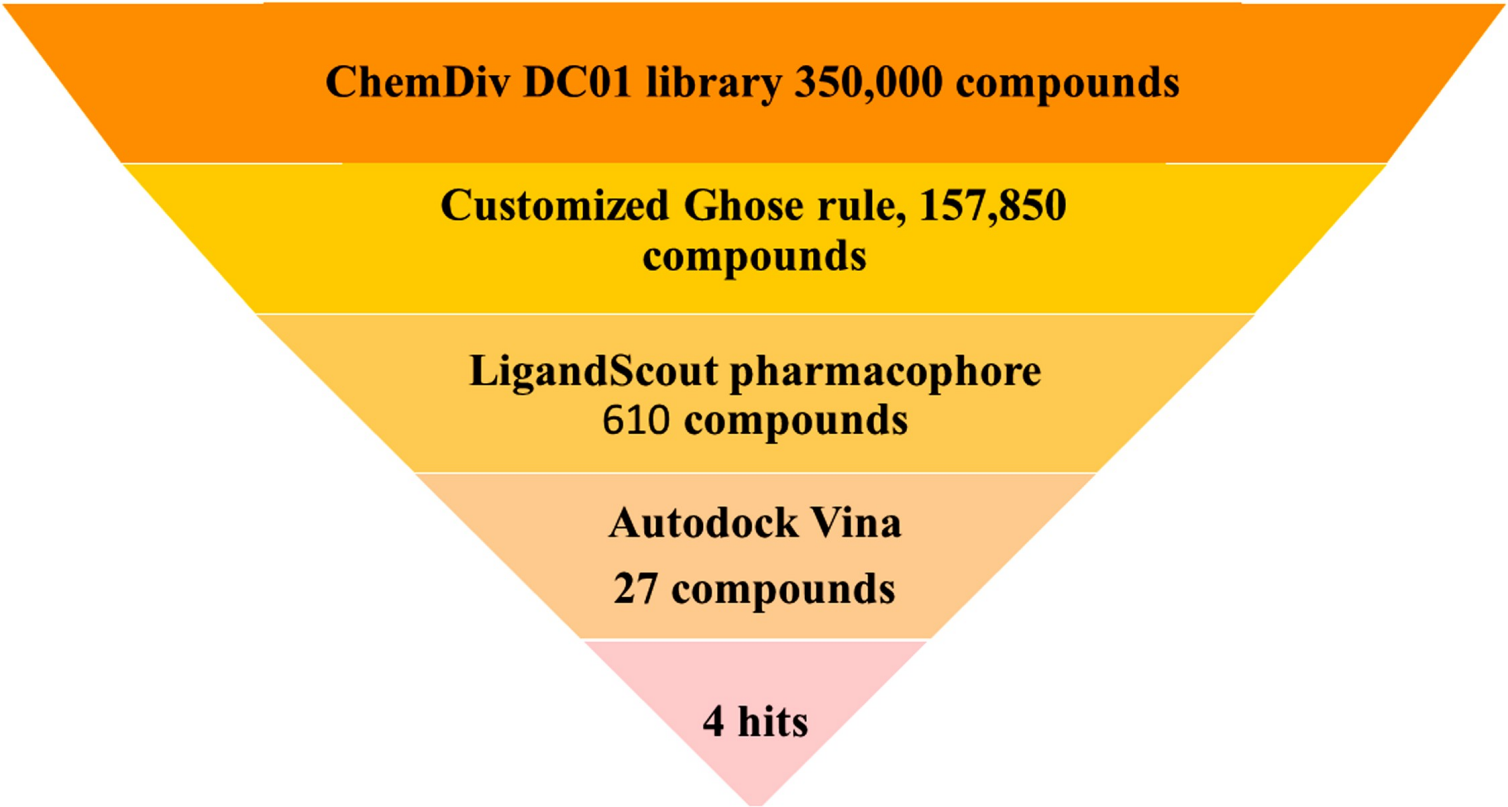
Schematic representation of the virtual screening process.

Fig 2 shows a flowchart of the VS procedure. The publicly available Discovery Chemistry, ChemDiv compound library (DC01), was used to obtain a combined dataset of 350,000 compounds. Selected candidates with good drug-like properties according to Ghose-rule[21] screening constituted a virtual library of 157,850 compounds.

### Ligand structure-based database screening

**Fig 3:**
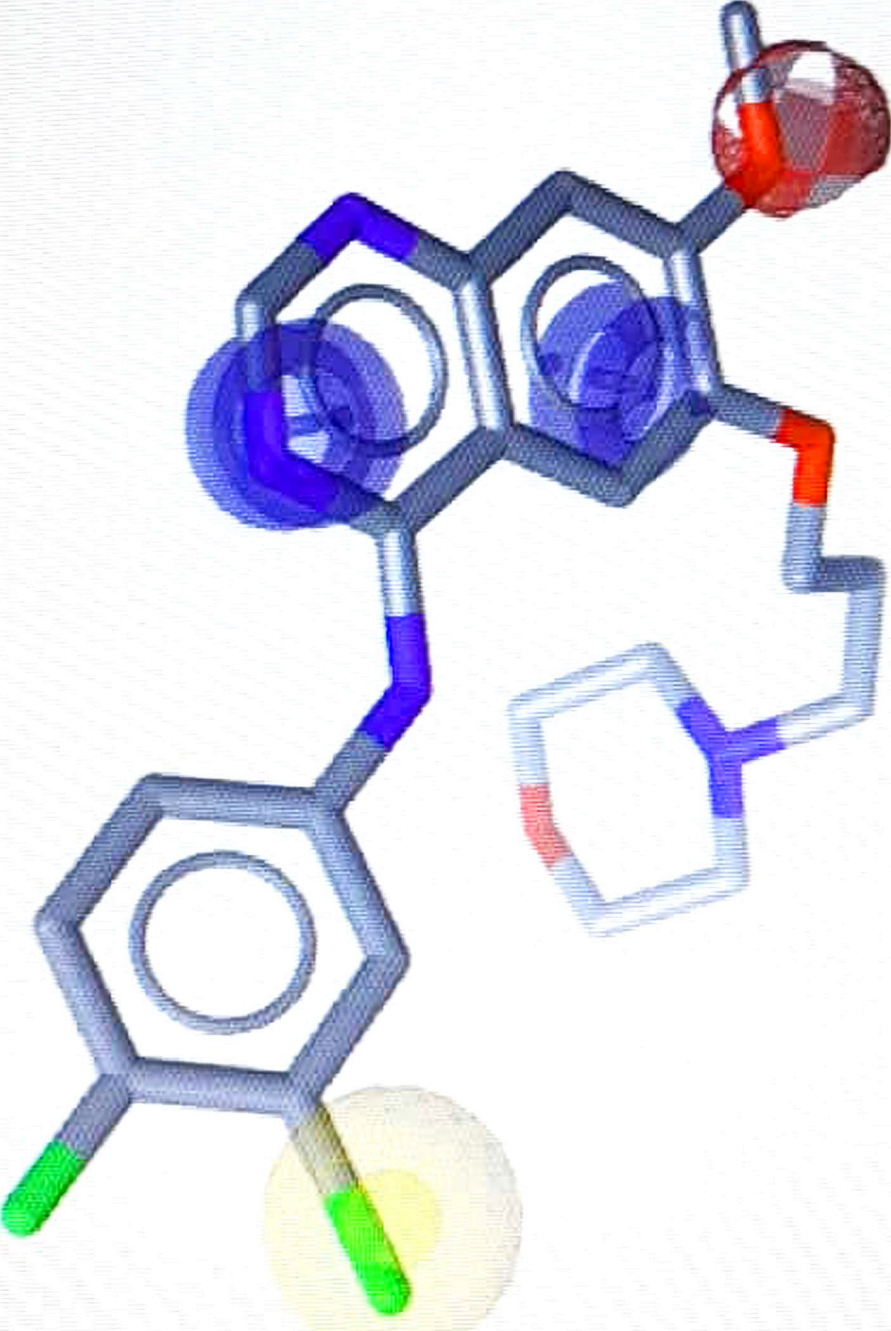
Ligand structure-based pharmacophore model of the gefitinib 4wkq.pdb structure. Hydrogen acceptor (red sphere), aromatic ring (blue circles) and hydrophobe (yellow sphere).

A pharmacophore is a spatial arrangement of functional groups or atoms that describes how a binder interacts with the binding site of a target protein. The pharmacophore theory assumes that the pharmacophores of compounds that share a binding site are identical. [21]. There are two pharmacophore modeling methods based on the knowledge available regarding ligands and receptors. Ligand-based methods involve the extraction of a common 3D-arrangement of chemical characteristics from a known diverse set of compounds that have similar binding mode to a certain macromolecule structure. However, a structure-based protocol requires the 3D structure of the active site or a target–ligand complex. Using the 3D structure of the EGFR–gefitinib complex (4wkq) [20], the LigandScout program [22] was used to build a pharmacophore model by the survey of the paired chemical characteristics of the binding site and their spatial organization around the binders. The resulting pharmacophore model consisted of two aromatic rings, one hydrophobic ring, and one hydrogen acceptor (Fig 3). LigandScout analysis decreased the library set to 610 hits (Supplementary File 1) based on pharmacophore score value.

### Database search using docking studies

The processed ligand set was docked using a consensus-docking approach [23]. This strategy is both feasible and affordable. The root mean square deviation (RMSD) between AutoDock Vina1.1.3 [24] and Autodock 4.2 [25] binding modes (RMSD ≤2) predicted for each complex yielded a small collection of 27 molecules with high scores, 4 of which had top docking scores in both Autodock Vina1.1.2 (−10.8: −9.5 Kcal^−1^) and Autodock 4.2 (−11.4: −9.28 Kcal^−1^) (Supplementary File 2) and were selected. The 2D structures, zinc ID numbers, and molecular weights of the four hit compounds are listed in Table 1. The hit compounds were hybrids of [1,2,4]triazolo[1,5-a]pyrimidin-7-one and a 2-(4-substitutedpiperazin-1-yl)-2-oxoethyl fragment.

**Table 1:** Results of the docking studies of the four selected hit molecules with the EGFR-TK protein.

ZINC ID / IUPAC name	Chemical formula (MWt.)	Docking energy (Kcal mol^-1^)			H-Bonds	π-Cation interactions	Hydrophobic interactions
		Autodock Vina1.1.2	Autodock 4.2	Estimated IC50 (nM)			
ZINC21802765 5-methyl-2-(2-methylphenyl)-4-[2-oxo-2-(4-phenylpiperazin-1-yl)ethyl]-4H,7H-[1,2,4]triazolo[1,5-a]pyrimidin-7-one	C_25_H_26_N_6_O_2_ (442.51)	-10.8	-11.4	787.87	Met793	Lys745	Val726, Ala743, Lys745, Thr790, Met793, Leu844,
ZINC21802749 5-methyl-2-(2-methylphenyl)-4-[2-(4-methylpiperazin-1-yl)-2-oxoethyl]-4H,7H-[1,2,4]triazolo[1,5-a]pyrimidin-7-one	C_19_H_22_N_6_O_2_ (380.2)	-10.5	-11.1	674.28	Met793	Lys745	Val726, Ala743, Lys745, Thr790, Leu792, Met793, Leu844
ZINC21802742 4-{2-[4-(4-fluorophenyl)piperazin-1-yl]-2-oxoethyl}-5-methyl-2-(2-methylphenyl)-4H,7H-[1,2,4]triazolo[1,5-a]pyrimidin-7-one	C_25_H_25_FN_6_O_2_ (460.5)	-10.3	-10.05	446.41	Met793	Lys745	Val726, Ala743, Lys745, Met766,Thr790, Met793, Asn842, Leu844
ZINC21802768 4-{2-[4-(2-methoxyphenyl)piperazin-1-yl]-2-oxoethyl}-5-methyl-2-(2-methylphenyl)-4H,7H-[1,2,4]triazolo[1,5-a]pyrimidin-7-one	C_25_H_26_N_6_O_2_ (472.55)	-9.5	-9.28	361.43	Met793		Leu718, Val726, Val726, Lys745, Leu792, Met793, Leu844, Thr854, Asp855

### Binding conformation of the hit compounds

**Fig 4:**
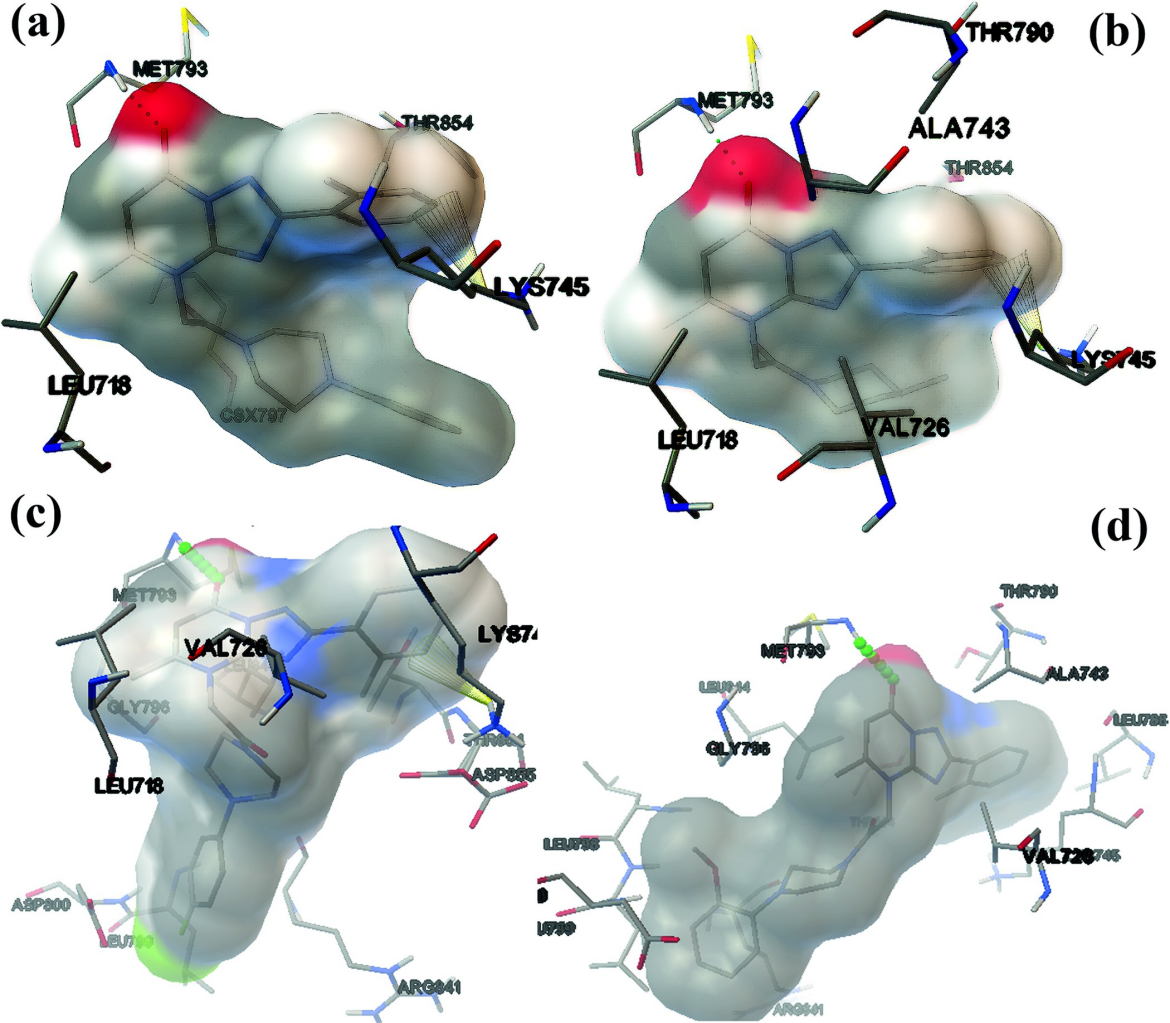
Autodock results of EGFR-TK with ZINC21802765 (a); ZINC21802749 (b); ZINC21802742 (c); ZINC21802768 (d). Hydrogen bonds in dotted lines; π-Cation interactions in yellow cone shape.

The selected hit compounds, ZINC21802765, ZINC21802749, ZINC21802742, and ZINC21802768 (Table 1), had a common scaffold skeleton, 2-phenyl-4H,7H-[1,2,4]triazolo[1,5-a]pyrimidin-7-one, which plays main role in the binding of ligands to proteins. For compounds ZINC21802765, ZINC21802749, and ZINC21802742, the pyrimidinone oxygen atom engaged in hydrogen bonding with Met793 and explored π–cation interactions the π-system of phenyl ring to the positive amino group of Lys745, while hydrophobic contacts were present between the 4-[2-oxo-2-(4-phenylpiperazin-1-yl)ethyl moiety and Leu718, Cys797, and Val726 in the central hydrophobic region. In contrast, compound ZINC21802768 exhibited a hydrogen bond to Met793 and hydrophobic interactions to the hydrophobic side chains of the binding-site residues (Fig 4).

#### MD Simulations

**Fig 5:**
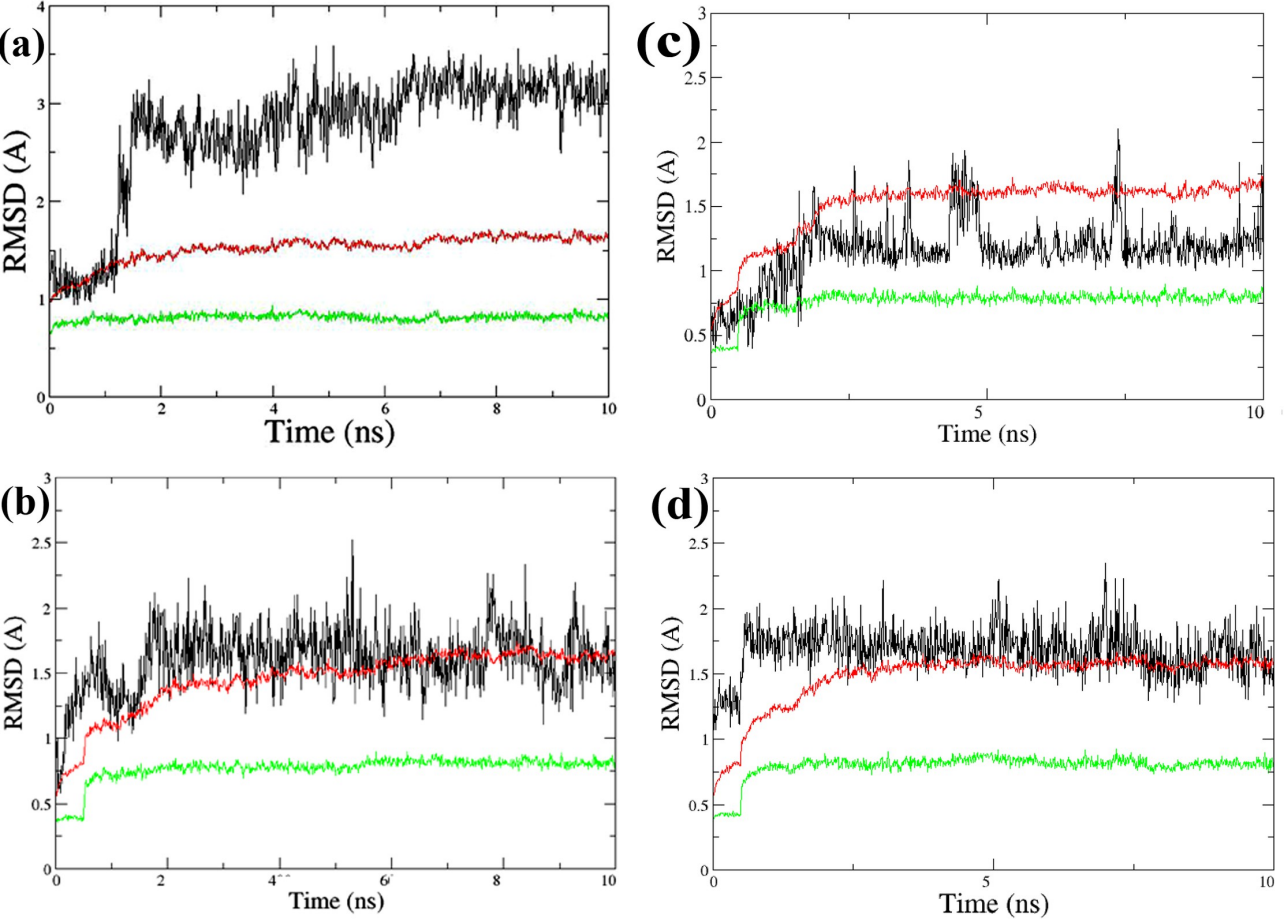
RMSD in MD simulations, showing protein backbones (green lines), protein all atoms (red lines), and ligands (black lines) for ZINC21802765 (**a**), ZINC21802749 (**b**), ZINC21802742 (**c**), ZINC21802768 (**d**).

Using AmberTools22 [26] EGFR-TK-docked ligand complexes of the selected compounds, ZINC21802765, ZINC21802749, ZINC21802742, and ZINC21802768, were used to compute the binding affinity and stability of the protein–ligand complex structures. All complexes were subjected to MD simulations, MM/GBSA [27] binding energy calculations, and molecular mechanics/Poisson–Boltzmann surface area (MM/PBSA) per-residue energy decomposition [28] to identify amino acid residues critical for in silico prediction of EGFR-TK-ligand binding affinity. It should be highlighted that docking studies, whether rigid or semi-flexible, provide a single snapshot of the interactions between ligands and proteins. Consequently, to better understand how the complex interaction profile is affected by protein structural variations and flexibility, the docked minimized complexes underwent 10 ns MD simulation time frames. The dynamic stabilities were determined using the RMSD changes during the MD simulations. Throughout the last 4 ns, the protein and ligand structures of the four complexes (Fig 5) equilibrated, with no apparent RMSD fluctuations.

**Fig 6:**
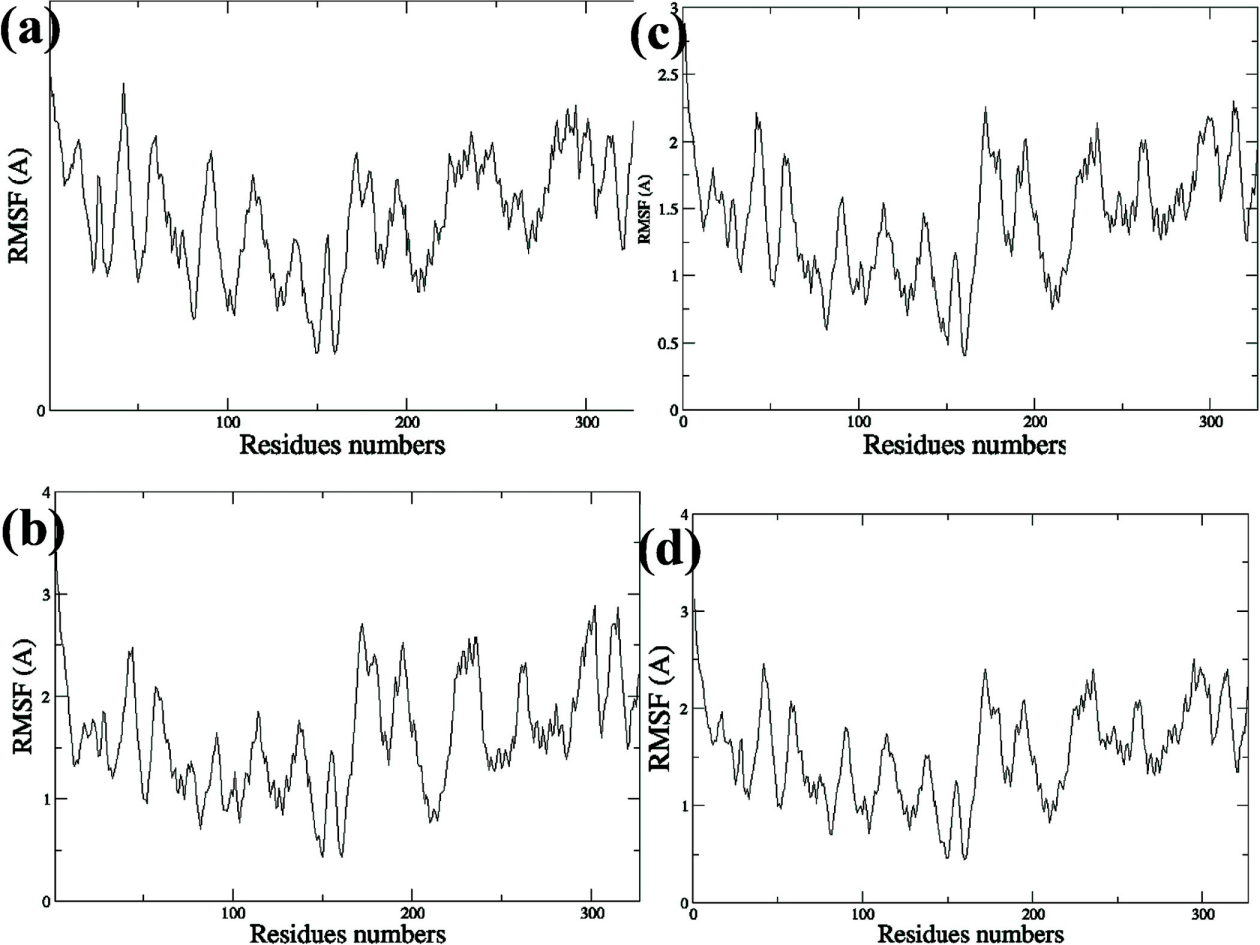
Per-residue root mean square fluctuation of EGFR-TK–ligand complexes over all simulations times. Protein complex with ZINC21802765 (**a**), ZINC21802749 (**b**), ZINC21802742 (**c**), ZINC21802768 (**d**).

The per-residue root mean square fluctuation of EGFR-TK–ligand complexes over the last 4ns simulation period shows the least flexible residues numbers 50–150 (original xray numbers 743–853) that suggest the great impact of these residues on the binding of the EGFR-TK receptor to the ligands (Fig 6).

**Fig 7:**
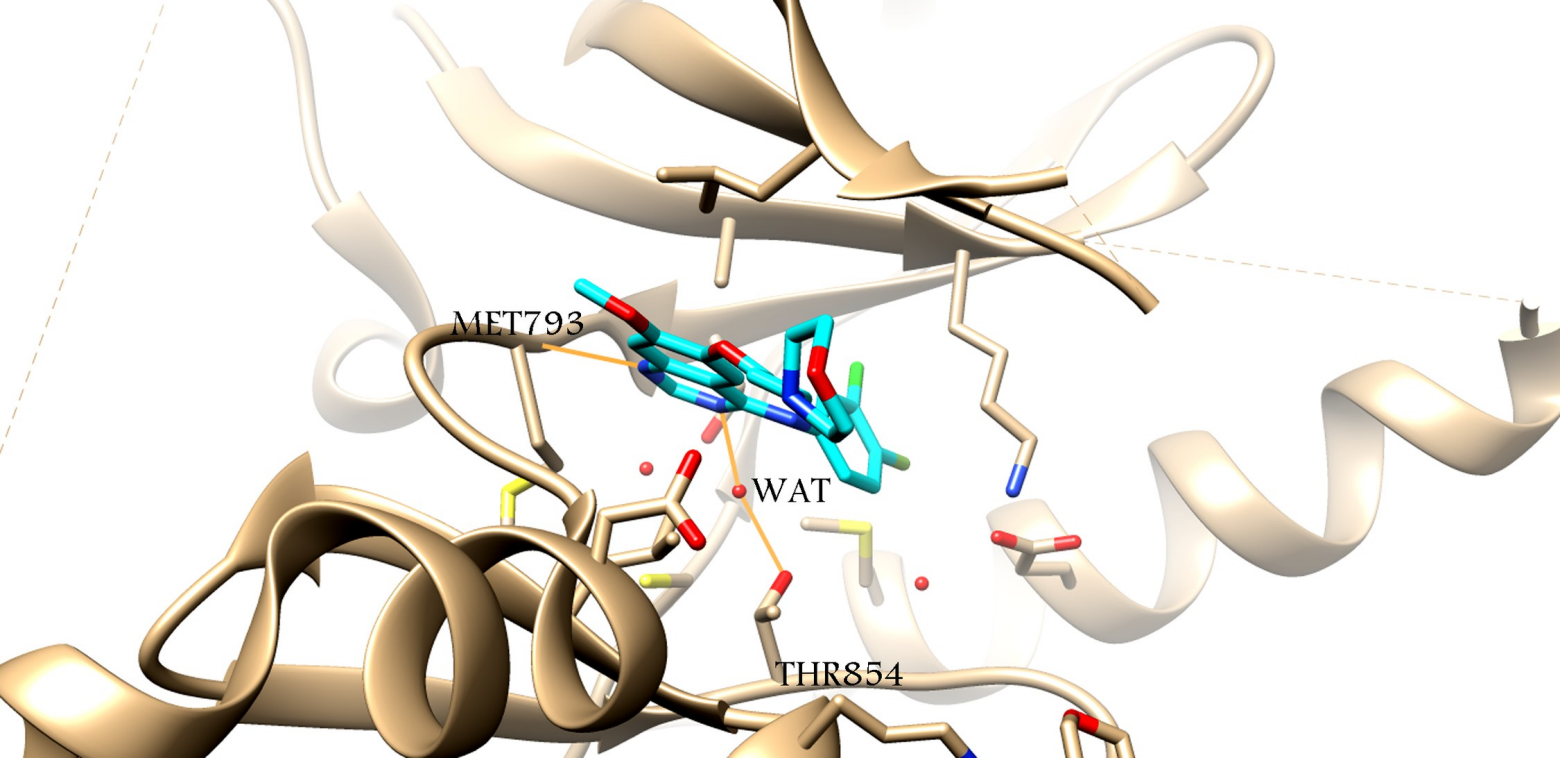
X-ray structure of EGFR-gefitinib (4wkq). Gefitinib structure (cyan stick), amino acids side chains (beige sticks), water molecules (red spheres).

The analysis of the crystal structure of EGFR-gefitinib (pdb id: 4wkq) revealed a contact distance to the side chain of Thr854 and a hydrogen bond bridge that connected the nitrogen atom of the pyrimindine ring of gefitinb to the Thr854 side chain via a water molecule Fig 7. According to recent reports, erlotinib-induced tyrosine phosphorylation inhibition was eliminated by the EGFR T854A mutation, leading to the development of erlotinib drug resistance [29]. Thr854 is found in the EGFR activation loop [30]. It may be possible to achieve more kinase selectivity in this binding form by combining interaction with the Met790 side chain with a hydrogen-bonding connection with Thr854 [31].

**Fig 8:**
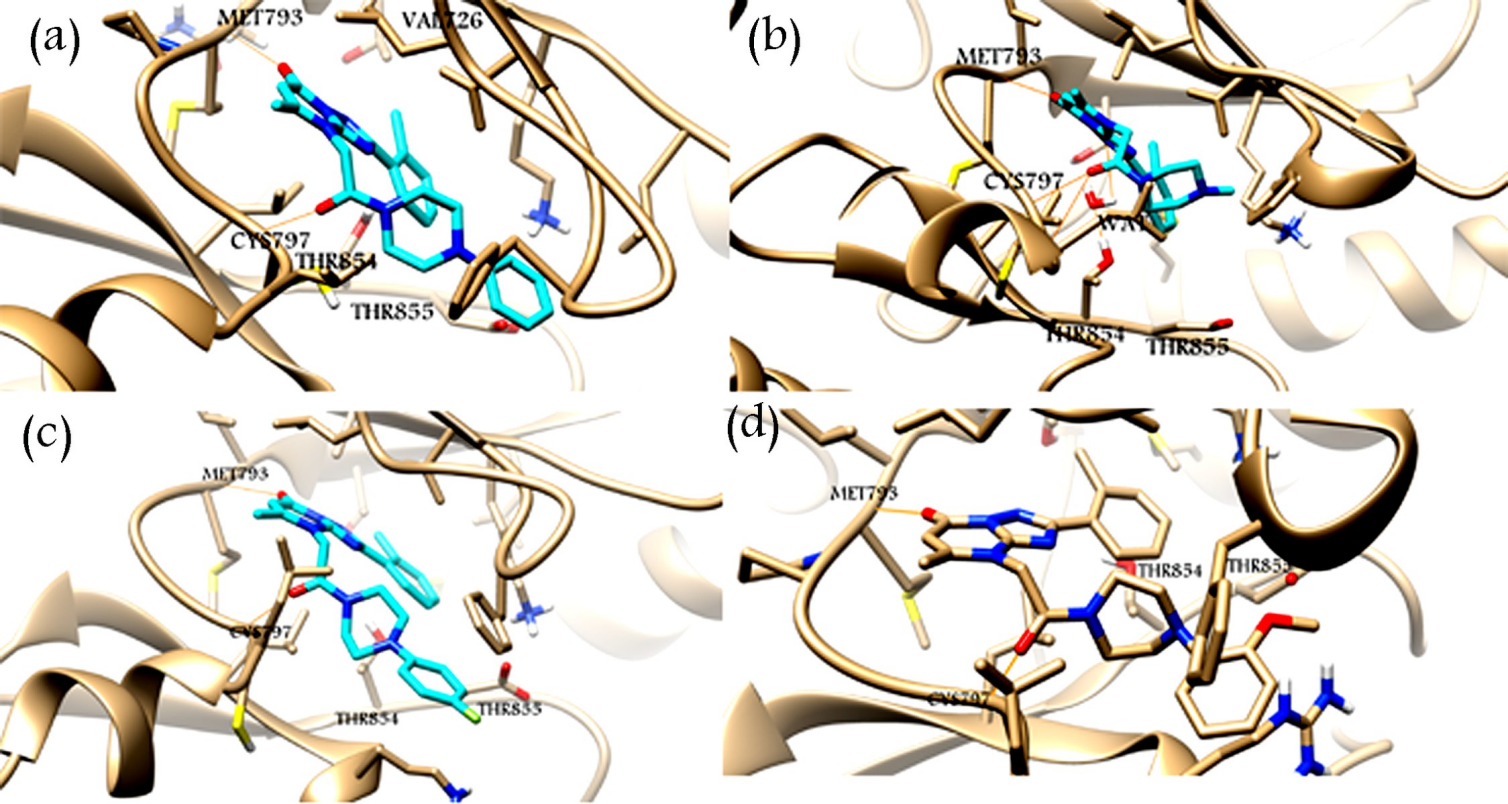
Average structures of EGFR-ligand complexes obtained during the final snapshots (4 ns) of MD simulations. The ligands are cyan colored sticks of ZINC21802765 (a), ZINC21802749 (b), ZINC21802742 (c) and ZINC21802768 (d).

In our study, the program for processing coordinate trajectories and data files, CPPTRAJ [32], showed the hydrogen bonds with high occupancy for the four simulation systems during the final 4 ns equilibration time frame, as shown in Table 2 and Fig 8. For the ZINC21802765–EGFR-TK complex Fig 8A, the amino acid residues Met793 and Cys797 formed a very stable hydrogen bond with the ligand, with occupancies of 95% and 84%, respectively. For ZINC21802749–EGFR-TK Fig 8B, amino acid residues Met793 and Cys797 formed stable hydrogen bonds with the ligand, with 86% occupancy. Moreover, ZINC21802749 explored hydrogen bond bridge (same hydrogen bond profile of gefitinib xray structure) between the nitrogen atom of its triazole ring to Thr854 side chain via a water molecule that suggests its significant EGFR-Tk inhibitor activity and potential selectivity. On the other hand, the remaining three compounds ZINC21802765, ZINC21802742, ZINC21802768 failed make hydrogen bond bridge between the nitrogen atom of its triazole ring to Thr854 side chain via a water molecule. This is could attributed to the bulk un(substituted)phenyl ring attached to piperazine nitrogen that increase the whole volume of the molecule that inside the ATP binding pocket that prevent the structure to orient near Thr854 at a distance enough for water-mediated bridge hydrogen bonding.

For ZINC21802742–EGFR-TK Fig 8C, the amino acid residues Met793 and Cys797 showed strong hydrogen bonds with the ligand, with occupancies of 83% and 77%, respectively. For ZINC218022768–EGFR-TK Fig 8D, the amino acid residues Met793 and Cys797 showed moderate hydrogen bonding with the ligand, with occupancies of 52% and 53%, respectively. The 2-methoxyphenyl moiety in ZINC218022768 structure was oriented between the side chains of Arg841 and Phe723. This orientation and the electronic effect of the methoxy group (positive inductive and mesomeric effects) may have a role in the weak inhibitor activity of the compound and it is suggested for further study and consideration.

#### Binding free energy estimation

Using 100 snapshots obtained from the final simulation trajectories, the binding efficiencies of the hit compounds were estimated using the MM/GBSA method (Table 2). The most active compounds, ZINC21802765 and ZINC21802749, showed preferred binding energies of −63.4480 and −61.5184 kcal mol^−1^ respectively. For all ligands, the vdW interaction energy values were two to three times those of the electrostatic interactions. The results in accordance with prior research on a number of small-molecule therapeutic kinase inhibitors, including olmutinib, lapatinib, and icotinib [33, 34].

**Table 2:** Binding free energy results of MM/GBSA calculations (kcal mol^−1^ ^a^).

Compounds	Hydrogen bond (occypancy %)	ΔG_vdw_	ΔG_elec_	ΔG_polar_^b^	ΔG_surf_^c^	Δ_GMMGBSA_
ZINC21802765	Cys797 (95.8) Met793 (84.0)	-60.5632	-29.7624	34.0888	-7.2112	-63.4480
ZINC21802749	Cys797 (86.6) Met793 (85.0)	-60.1177	-29.3884	34.9215	-6.9337	-61.5184
ZINC21802742	Cys797 (83.8) Met793 (77.2)	-56.6452	-23.6452	28.0661	-6.4519	-58.6762
ZINC21802768	Cys797 (52.4) Met793 (53.6)	-52.3461	-19.6195	23.3532	-6.1635	-54.7760

^a^ Average of 1000 frames

^b^ Whole electrostatic contribution: ΔG_elec_ = ΔG_electrostatic_ + ΔG_polar_

^c^ Whole nonpolar contribution: ΔG_np_ = ΔG_vdw_ + ΔG_surf_

### Per-residue free energy decomposition

The MM/PBSA analysis was used in identifying the amino acid residues crucial for effective binding to the EGFR-TK active site [35,36]. For the active hit compounds, EGFR-TK-ZINC21802765 and EGFR-TK- ZINC21802749 complexes, the residues Val726, Leu792, Met793, Gly796, Cys797, Leu798, and Leu844 showed decomposition energy values in the range of–-2.025 to–-3.194 kcal mol^−1^ and -2.082 to -3.595 194 kcal mol^−1^ respectively. For the partial active compound, EGFR-TK- ZINC21802742 complex, the residues Leu792, Met793, Gly796, Cys797, and Leu844 showed decomposition energy values in the range of–-2.0 to–-2.985 kcal mol^−1^. For the weak active hit compound, EGFR-TK- ZINC21802768 complex the residues Gly796 and Leu798 showed decomposition energy values in the range of–-2.013 to -2.45 kcal mol^−1^ (Table 3).

**Table 3:** Results of per-residue energy decomposition analysis (kcal mol^−1^).

EGFR-TK residues	Val726	Leu792	Met793	Gly796	Cys797	Leu798	Leu844
**ZINC21802765**	-2.562	-2.025	-2.753	-3.194	-2.057	-2.471	-2.641
**ZINC21802749**	-2.845	-2.948	-2.237	-2.773	-2.455	-2.082	-3.595
**ZINC21802742**	-2.0	-1.024	-2.418	-2.088	-2.179	-1.498	-2.985
**ZINC21802768**	-1.384	-1.805	-1.463	-2.450	-1.030	-2.130	-1.158

### Biological evaluations

#### EGFR phosphorylation inhibition

**Fig 9:**
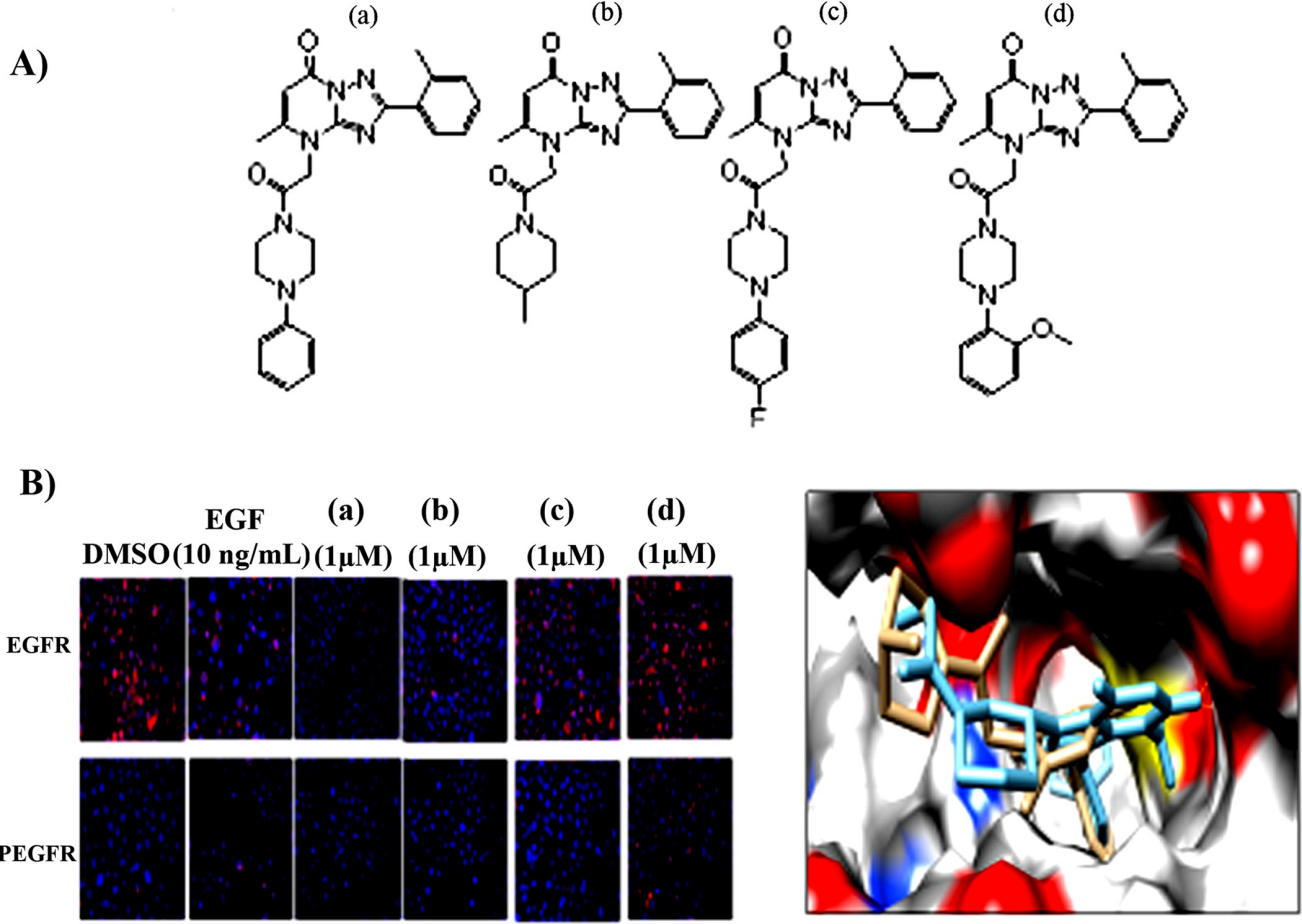
**A**) Hit compounds ZINC21802765 (a), ZINC21802749 (b), ZINC21802742 (c), and ZINC21802768 (d). **B**) Staining of H1975 cells with EGFR and PEGFR. Compounds a, b, and c inhibit the autophosphorylation of EGFR induced by EGF (10 ng/mL) treatment. H1975 cells were stained with primary antibodies against EGFR/p-EGFR (magenta) followed by secondary antibodies and DAPI (red). Thereafter, the constituted EGFR and PEGFR proteins and localization were determined by immunofluorescence assay.

Epidermal growth factor receptor (EGFR) is an oncogenic tyrosine kinase receptor that driving the initiation and progression of non-small-cell lung cancer (NSCLC) [37]. First-generation EGFR-tyrosine kinase inhibitors (TKIs) such as gefitinib and erlotinib reversibly bind to the ATP cleft within the EGFR kinase domain to block auto phosphorylation of EGFR [38]. Several autocrine growth factors, including as EGF and TGFα, promote EGFR. Here, we evaluated the anti-EGFR activity of the hit compounds by inhibiting EGF-induced phosphorylation. The activity of hit compounds was examined by inhibiting EGF-induced EGFR phosphorylation and confirmed by immunofluorescence assay in H1975 cells. Staining of untreated and compound-treated cells by total and phosphorylated EGFR antibodies, showed complete inhibition of EGFR phosphorylation by ZINC21802765 and ZINC21802749. Partial inhibition was detected with ZINC21802742 and no activity was observed with ZINC21802768 treatment (Fig 9). Interestingly, both ZINC21802765 and ZINC21802749 inhibited the total EGFR and phosphorylated expression which, indicated full coverage on the drug target. Compounds ZINC21802765 and ZINC21802749 with complete inhibition of wild type EGFR possess a phenyl or methyl group respectively at the 4-position of piperazine moiety, these two compounds possess phenyl or methyl group respectively. On the other hand, compounds ZINC21802742 and ZINC21802768 with either partial or no inhibitor activity respectively of wild type EGFR possess p-fluorophenyl or o-methoxyphenyl group at the the 4-position of piperazine moiety. These results suggest the smaller the group at 4-position of piperazine the better the inhibitory activity of the compound.

#### Induction of apoptosis

**Fig 10:**
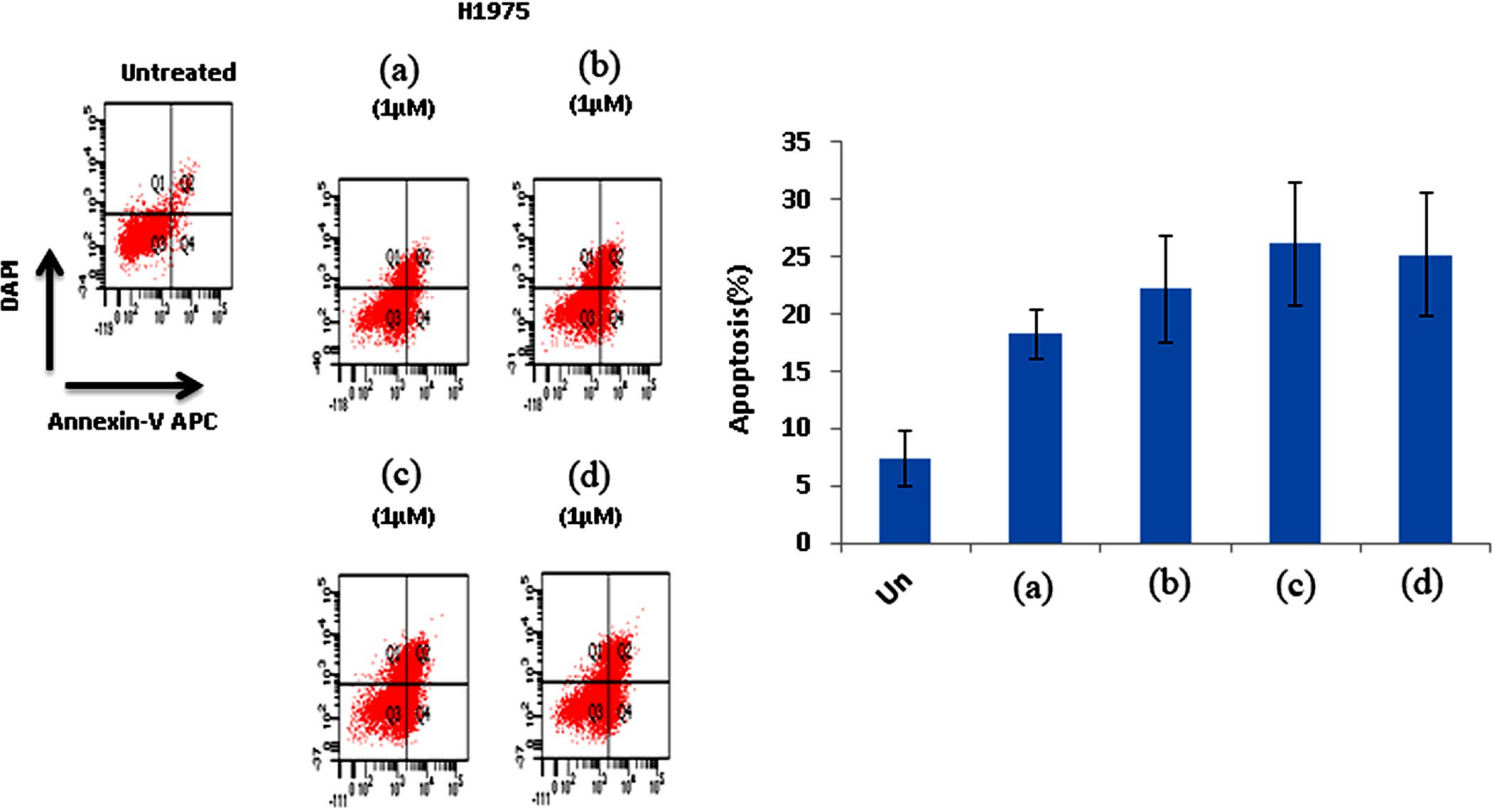
Apoptosis induction effect of compounds. The cells were collected and then stained with annexin V–APC/DAPI. The percentage of cells underwent to apoptosis was then analyzed on a LSRII Flow Cytometer. One of the three representative experiments using different cell preparations was only shown. The values represent mean ± SEM (n = 3). Compounds are ZINC21802765 (a), ZINC21802749 (b), ZINC21802742 (c), and ZINC21802768 (d).

**Fig 11:**
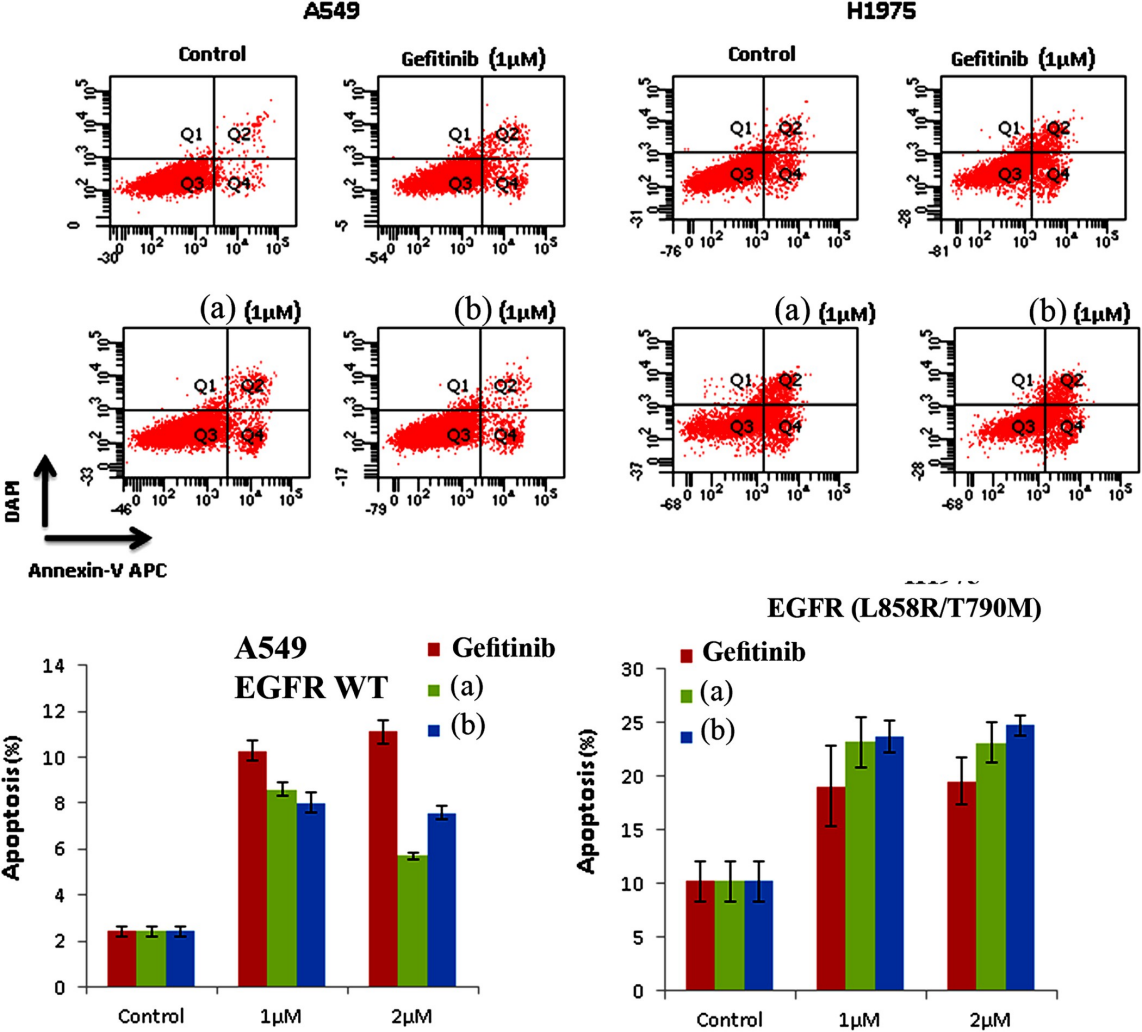
Selective apoptosis induction effect of compounds on H1975 cells harboring the EGFR L858R/T790M double mutation vs A549 cells that harboring wild-type EGFR compared with EGFR inhibitor reference drug gefitinib. The percentage of cells that underwent apoptosis was then analyzed on LSRII Flow Cytometer. The values represent mean ± SEM (n = 3) *; p < 0.05 compared to control by Student’s t-test. Compounds are ZINC21802765 (a), ZINC21802749 (b).

We first sought to investigate whether the inhibition of hit compounds on EGFR phosphorylation will lead to inhibit the survival of lung cancer cells. Therefore, we assessed the efficacy of hit compounds induced apoptosis on H1975 cells as a marker of cell killing and decreased cell survival. For this purpose, cells were treated with the indicated hit compounds in 1μM for 48 hrs, thereafter the percentage of cells underwent apoptosis was determined by flow cytometry. Treatment with compounds ZINC21802765, ZINC21802749, ZINC21802742, and ZINC21802768 induced efficient apoptosis as shown in Fig 10. Hit compounds showed increase in apoptosis percentage by 20.6,22,25 and 26% compared with 10% in mock treated cells. Unlike the inhibition of EGFR phosphorylation, the induction of apoptosis was shown to be independent of EGFR inhibition activity. Furthermore, effective response and good clinical outcomes of EGFR inhibitors, enable their use as the first-line setting for patients with advanced NSCLC harboring activating EGFR mutation (a deletion in exon 19 or the L858R mutation in exon 21) [39, 40]. However, NSCLC patients initially response to these EGFR-TKIs almost invariably develop drug resistance [41], which commonly arise through the acquisition of a second-site mutation (T790 M) within EGFR, or via activation of compensatory signaling pathways that bypass receptor and restore downstream oncogenic signaling [42]. Therefore, we sought to evaluate the induction of apoptosis using H1975 cells harboring the EGFR L858R/T790M double mutation and A549 cells that harboring wild-type EGFR. ZINC21802765 and ZINC21802749 the most active hits were used and compared with the EGFR inhibitor reference drug Gefitinib. The treatment showed a persistent apoptosis-inducing effect on mutant EGFR H1975 cells and increased the percentage of apoptosis by 20.5 and 22%, receptively. In higher concentration by 2μM, the apoptosis percentage was increased by 21 and 25.8%, respectively. Both hit compounds are more effective than gefitinib by at least one fold in inducing cell killing toward mutant EGFR H1975 but not on wild-type A549 cells as shown in (Fig 11). Taken together, the biological evaluation results of hit compounds showed inhibition of EGFR and selective apoptosis-induced effects on mutant cells, rather than on wild-type lung cancer cells.

## Conclusions

In conclusion, the study utilized a ChemDiv dataset to identify drug-like compounds for EGFR-TK inhibition. Through various computational methods, four hit compounds were identified and their interactions with EGFR-TK were analyzed. The most active compounds showed strong inhibition of EGFR and selective effects on mutant cells, suggesting their potential as therapeutic agents for lung cancer. We anticipate that our current research into novel and specific EGFR-TKIs using the ChemDiv database will be helpful in identifying new compounds with therapeutic potential against lung cancer.

## Supporting information

S1 File Results of the virtual screening using LigandScout program. (XLS)

S2 File Results of the virtual screening using Autodock Vina and Autodock 4.2 programs. (XLSX)
